# Brain substrates explain differences in the adoption and degree of financial digitalization

**DOI:** 10.1038/s41598-020-74554-3

**Published:** 2020-10-15

**Authors:** Santiago Carbo-Valverde, Juan A. Lacomba-Arias, Francisco M. Lagos-García, Francisco Rodriguez-Fernandez, Juan Verdejo-Román

**Affiliations:** 1 CUNEF, Bangor University, Bangor, UK; 2 Funcas, Madrid, Spain; 3 Department of Economics and Mind, Brain, and Behavior Research Center (CIMCYC), University of Granada, Granada, Spain; 4 Zayed University, Dubai, UAE; 5 Laboratory for Cognitive and Computational Neuroscience (UCM - UPM) and Experimental Psychology Department, School of Psychology, UCM, Madrid, Spain

**Keywords:** Neuroscience, Physiology

## Abstract

This study analyzes neural responses connected to trust and risk to explain financial digitalization decisions. It shows that brain responses distinctively inform differences in the adoption of digital financial channels that are not shown by any other sociodemographic or behavioral indicators. From a methodological standpoint, the study explores if usage patterns of digital financial channels and instruments are associated with psychological and biological indicators; it uses functional magnetic resonance imaging (fMRI) to investigate whether financial digitalization decisions are linked to the evoked brain response to the safety associated with video images of financial transactions through digitalized and non-digitalized channels; it conducts trust and risk neuro-experiments to identify their impact on financial digitalization decisions and it analyzes whether brain structure is linked to financial digitalization behavior. The findings suggest that high and low frequency users exhibit differences in brain function and also in volume and fractional anisotropy values. A higher frequency of use of financial digital financial services is associated with higher brain activation linked to insecurity (lower safety neural evoked responses during the video task and an altered white matter microstructure of the cingulum). Additionally, high frequency users of digital financial channels exhibit enhanced activation of brain areas linked to emotional processing during the trust game. These findings have important implications for the design of public policies to enhance financial inclusion through technology and the segmentation and service distribution strategies of private financial institutions.

## Introduction and motivation

Financial decisions have been identified to be bounded in terms of rationality. This may have a significant impact on wealth and equality. Digital channels have emerged as alternatives to individuals to make financial decisions. Financial digitalization may improve the efficiency and speed of retail financial services^1^. At the same time, digitalization alters the way individuals gather information and make financial transactions based on criteria such as the trustworthiness and perceived risk of digital channels and also personal traits such as impulsiveness or sensitivity to reward and punishment.

A number of studies have shown that due to cognitive constraints and a low average level of financial literacy, many savings, investing and borrowing decisions of individuals violate sound financial principles^2–7^. Relatedly, a fundamental characteristic of digital channels is the online (non-human) nature of the interaction, while there is a human interaction attached to offline services. Hence, introducing the digital dimension to financial decision-making is not trivial, as people differ substantially in the way they undertake their financial digitalization choices. In this study, we focus on trust and risk dimensions of financial digitalization decisions, although we also control for other factors potentially interacting with trust and risk, including impulsiveness and reward mechanisms.

It has been shown that although trust plays a role in most economic transactions, it is even more relevant in digital settings^8,9^. There have been some large studies analyzing the relationship between trust and the activation of brain regions in human-based offline settings, mainly conducted through trust neuro-experiments^10–13^. More recently, there have also been some analyses on trust and related brain activations in online interactions^14,15^. The main findings of these studies demonstrate that brain regions such as the striatum, cingulate and prefrontal structures enhance digital or online trustworthiness, whereas the amygdala and the insular cortex are more activated in discreditable or malevolent economic situations.

As most financial decisions entail risk, a change in the channels that define the environment for the transaction and information exchange seems important. Most of the previous research regarding neural analyses of risk corresponds to individual investor’s trading in stock and debt markets and the relationship between risk and return. Evidence has been produced on the role of the activity in the anterior insula during the assessment of risky versus safe choices in an investing task^16^. Earlier analyses also suggest ventral striatum activity is associated with riskier investment profiles, while activity in the anterior insula is associated with low risk investment profiles^17–22^. Similarly, activity in the anterior insula has been proven to be related to risk-related sensations of uncertainty and pain^23^.

In addition to trust and risk processing, other factors related to financial behavior are impulsiveness and reward mechanisms. In certain environments, unconscious brain processes drive impulsive behavior^24^. The brain regions involved in this process are mostly nested within the so-called “reward system” that registers stimulus and deception related to expectations for certain events and processes^25^. Earlier fMRI studies have also shown that the striatum is involved with the processing and anticipation of rewards^26,27^. Digital channels incorporate potential reward mechanisms (e.g., time saving, lower fees) compared to traditional (physical) channels and may also stimulate different impulsiveness responses than offline channels. It has been also shown^28^ that the nucleus accumbens, the sublenticular extended amygdala (SLEA), and the hypothalamus are involved in the processing of the prospects of monetary or economic rewards in a similar manner to processing reactions to tactile stimuli, gustatory stimuli, or euphoria-inducing drugs. Similarly, medial-frontal regions also contribute to mental states that participate in high-level decisions, including economic choices^29^. In the context of human versus non-human economic interactions, there is a trade-off between known facts and uncertainty, and this trade-off affects the risk-reward mechanism^30^. The fMRI reveals these human versus non-human preferences predict brain activation associated with decision making. Ambiguity preference was related to the lateral prefrontal cortex, and risk preference predicted the activity of the posterior parietal cortex. The question of whether the choice of digital financial channels entails uncertainty for individuals and the activation of brain-related trust or risk-reward mechanisms remains unexplored.

While a number of studies have dealt with how brain activity informs financial risk-taking behavior, little is known about its impact on financial digitalization decisions. This is particularly relevant if financial digitalization choices are not only motivated by revealed risk and trust attitudes towards digital financial channels, but also by brain activation patterns across heterogeneous groups in terms of financial digitalization adoption. Thus, we aim to explore the trust and risk neuropsychological factors and brain substrates that underpin financial digitalization decisions in adults with different patterns of digitalization adoption. The identification follows four steps. We first explore if adoption patterns were associated with psychological traits that had been previously linked to financial behavior (including impulsiveness and sensitivity to reward). Second, we use functional magnetic resonance imaging (fMRI) to investigate if financial digitalization decisions were linked to the evoked brain response to the safety associated with video images of financial transactions, including digitalized and non-digitalized channels. Third, we conduct trust and risk neuro-experiments to identify their impact on financial digitalization decisions. Finally, as a general check and comparison to other studies, we explore if brain structure could distinctively explain financial digitalization behavior.

Based on the findings of previous studies and the differences found between human and non-human financial channels, a first hypothesis—that serves as a first filter before the trust and risk tests—is that participants with higher digitalization adoption will show higher levels of impulsiveness and sensitivity to reward. As a second hypothesis, we would also expect that participants with higher digitalization adoption will show higher activation on the prefrontal, cingulate, and striatum cortexes. Specifically, we analyze the neural responses of study participants while they watched financial transaction videos and also while they played trust and risk games. As a third hypothesis—and a comparison with other studies in different economic settings—we would also expect to find structural differences in the prefrontal and reward areas between groups.

## Methods

### Participants

One hundred and twenty-one healthy adults, aged between 18 and 33, participated in this study. They were recruited through media advertisements, and all of them completed an online survey on their financial habits. They were classified into three groups according to the frequency of use of online financial services: 40 with weekly use (high frequency use, HFU), 40 with monthly use (low frequency use, LFU) and 41 that never or almost never use (NU). Magnetic Resonance Imaging (MRI) contraindications (e.g., claustrophobia, ferromagnetic implants) or abnormalities in the MR images were exclusion criteria.

All participants had normal or corrected-to-normal vision. The study was approved by the Ethics Committee for Research in Humans of the University of Granada (Spain) (Approval code: 717) and was conducted in accordance with the Declaration of Helsinki. All participants signed written informed consent.

### Procedure

An online survey was published to ask the general population about their financial habits. The main structure of the survey followed that of the Survey of Consumer Payment Choice conducted by the Federal Reserve Bank of Boston. However, our survey incorporated comprehensive information about consumers’ digital preferences. Furthermore, the survey included information about a set of factors that, based on theoretical foundations for technology acceptance, explains the adoption and use of digital channels (e.g., perceived usefulness, cost, complexity, convenience, and risk). Controlled quotas for a representative sample of the population were established based on age, sex, and location. All participants in the survey were offered the opportunity to participate in a second session in which some tests were administered and a MRI session was held.

At the beginning of the MRI session, before scanning, a clinical psychologist with a master’s degree assessed tests and conducted trainings on tasks. At the end of the instructions, all subjects had to complete a questionnaire in order to verify that they understood the instructions. Afterwards, participants underwent the MRI scanning session, which included two fMRI tasks, a T1-weighted structural acquisition, and a diffusion tensor imaging (DTI). In the first fMRI task, participants had to watch some videos related to financial transactions, and the second task was a computer version of the trust and risk game. The MRI session lasted around one hour. At the end of the experiment, subjects were paid based on a publicly announced exchange rate of 25 points = 1 €.

### Tests administered before scanner

The Sensitivity to Punishment and Sensitivity to Reward Questionnaire (SPSRQ)^31^ is a 48-item questionnaire that comprises two subscales to measure the constructs of sensitivity to reward (SR) (24 items) and sensitivity to punishment (SP) (24 items). This questionnaire has demonstrated internal consistency, construct validity, and significant associations with brain systems relevant to reward and punishment.

The urgency, premeditation, perseverance, and sensation seeking scale (UPPS‐P) was also applied to obtain an impulsive behavior scale, using a brief Spanish version^32^. This questionnaire allows a multi‐dimensional assessment of impulsivity, including five different traits: (1) negative urgency; (2) positive urgency; (3) sensation‐seeking; (4) lack of premeditation; and (5) lack of perseverance. It has shown adequate psychometric properties (Cronbach's α values ranging from 0.61 to 0.81).

### fMRI tasks

#### Videos

We used 18 videos belonging to nine categories. Three of them referred to traditional financial transactions (i.e., withdrawing cash from an ATM and paying with cash or with a credit card), four of them referred to more recent digital transactions (i.e., an online bank transfer and paying with PayPal, a mobile phone, or a watch), and the remaining two were videos of pleasant and unpleasant animals. Each video lasted 15 s and was displayed twice to each participant. Immediately after each video, participants rated the intensity of the security they felt on a 1–4 number scale that appeared for 5 s (where 1 is “unsafe” and 4 is “extremely safe”). The total video task comprised 12 min.

#### Trust and risk games

In order to explore whether the frequency of use of digital financial channels is associated with interpersonal trust, we implemented a fMRI adaptation of the trust and risk games^33^.

##### Trust game

The game consisted of one investor and one trustee. Participants always played the investor’s role. Participants were told that they were always matched with a randomly selected different person in each trial (i.e., we used strangers matching protocol). To enhance the credibility and interpersonal appeal of the game, participants were told that the trustee was another participant in the research project who had been randomly selected from the pool of previous participants. In each trial, participants received an initial endowment of 12 points, and investors could send 0, 4, 8, or 12 points to the trustee. The transferred points were tripled by the experimenter. Then the trustee had the option to send any amount between zero and his or her total amount available back to the investor. Investors were told that trustees had already made a decision for each possible transfer. Then our participants made their decisions by pressing one of four buttons that we provided, which indicated the number of points to be transferred.

##### Risk game

Participants faced the same choices as in the trust game. However, participants were paired with a random computer mechanism in the role of trustee, and this was common knowledge for participants. If participants decided to transfer points, they knew that a computer would decide whether or not to return points. They were told that the probability of the computer returning points was based on the probability distribution generated by trustees’ decisions in the trust game. In this manner, participants faced the same probability of having points returned in both treatments, but in the risk game there was no room for interpersonal expectations such as trust, betrayal, or reciprocity.

Subjects played 12 trials of each game in a pseudorandomized order, for a total of 24 trials. At the beginning of each trial, participants watched a message in the screen for four seconds illustrating which game they were going to play. Then, they had eight seconds to decide how much they were going to transfer and press the appropriate button. We called this whole 12-s period the decision phase. After this phase, a new screen appeared for eight seconds, informing the participants that the trustees were making their decision. Finally, each trial finished with a fixation cross screen with a variable duration of 10–12 s. Participants did not receive any feedback regarding points returned by either trustee or computer in each trial. After the first 12 rounds, participants received information regarding the number of times that her or his pair, either person or computer, had returned points. At the end of experiment, participants received information about the total points obtained in each trial of both the trust and risk games.

In the trust game, if the investor transfers points to the trustee and the latter reciprocates, both participants end up with a higher amount of points. However, the trustee also has the option of violating the investor’s trust by not returning points. In this case, the investor loses all the points he or she sent to the trustee, an event that investors typically interpret as a betrayal of trust^34^. Since sending points is costly for the trustee, a selfish trustee will never reciprocate the investor’s trust because investor and trustee interact only once in the experiment.

The key difference between the trust and the risk games is that, while in the risk game the investor’s risk depends on a random mechanism, in the trust game, it arises from the uncertainty regarding a social interaction with a real person in the role of trustee. Experimental evidence shows that individuals are averse to being betrayed^35,36^. Therefore, in our experiment, whenever investors show a lower willingness to take risks in the trust game than in the risk game, it will be interpreted as “betrayal aversion”.

In both trust and risk games, the amount transferred was collected trial by trial. The mean amounts sent in the trust and risk games were computed. Additionally, in order to examine the aforementioned betrayal aversion, we aim to compare the transferred points in each game and calculate a betrayal aversion score by subtracting the amount transferred in the risk game minus amount transferred in the trust game.

### Imaging acquisition and preprocessing

Brain data was collected using a 3 T Magnetom Tim Trio scanner supplied by Siemens Medical Solutions (Erlangen, Germany). This scanner is equipped with a 32-channel receive-only head coil. During each functional task, we acquire T2*-weighted echo-planar imaging (EPI) sequences. The following parameters were used: Repetition time (TR): 2,000 ms; echo time (TE): 25 ms; flip angle: 80º; field of view (FOV): 238 mm; number of slices: 35; voxel size: 3.5 × 3.5 × 3.5 mm; gap: 0.7 mm; number of volumes: 390 and 410 for the trust and video tasks, respectively. All images were obtained axially and parallel to the AC-PC plane^37^.

Images for the structural analyses were obtained. In particular, a sagittal three-dimensional T1-weighted image and a diffusion tensor imaging sequence. The parameters provided to obtain the images were: For the TR: 2,300 ms for the 3D image; 3.1 ms for TE; 9º flip angle; 256 mm FOV; 208 slices; 0.8 × 0.8 × 0.8 mm voxel dimension. For DTI acquisition: TR: 9,400 ms; TE: 88 ms; FOV: 256 mm; 72 slices; 2.0 × 2.0 × 2.0 voxel dimension; 30 volumes with diffusion weighting (b = 1,000 s/mm^2^) and one volume without diffusion weighting (b = 0 s/mm^2^)^37^.

The software used to obtain the functional images was the Statistical Parametric Mapping (SPM12). SPM is made freely available to the [neuro]imaging community by the University College London (Welcome Department of Cognitive Neurology https://www.fil.ion.ucl.ac.uk/spm/) and it does not require permission to be used. SPM12 runs under Matlab R2017 (MathWorks, Natick, MA, USA). The preprocessing using SPM12 includes realignment to the first image of the time series, co-registration to the structural image of each participant, unwarping, slice-timing correction, outlier detection, normalization to an EPI template in the Montreal Neurobiological Institute (MNI) space. It also incorporates spatial smoothing by convolution with a 3D Gaussian kernel [full width at half maximum (FWHM) = 8 mm]^37^.

T1 image processing was conducted using the recon-all automated processing pipeline in Freesurfer (version 6.0). Cortical and subcortical volumes were automatically calculated based in the Destrieux atlas^38^ and the subcortical Freesurfer parcellation^39^.

Diffusion tensor images were preprocessed using FSL^40^ and included head motion and eddy-current induced artifacts correction, rotation of the gradient directions table, and brain extraction. Fractional anisotropy (FA) and mean diffusion (MD) maps were calculated using the dtifit function. The automated AutoPtx^41^ pipeline was used to run probabilistic tractography for some of the main system fibers in each individual. Complete details of the process are described elsewhere^42^.

All images were inspected for artifacts after acquisition. Outputs were also checked to discard outliers and incorrect processing.

### Statistical analyses

#### Behavioral analyses

Behavioral data was analyzed with the Statistical Package for the Social Sciences version 20 (SPSS; Chicago, IL). Differences between groups in demographic and neuropsychological variables (e.g., age, sex, SPSRQ scores, and UPPS-P scores) were tested using one-way ANOVAs and then by Tukey’s honestly significant difference (HSD) post-hoc tests. Each ANOVA analysis included one demographic or neuropsychological variable as the dependent variable and the group as the independent variable. Behavioral responses during the fMRI tasks (i.e., self-reported feelings of safety, amount transferred during the trust and risk games, and betrayal aversion scores) were compared between groups using two-sample Mann–Whitney tests. Additionally, the Wilcoxon signed-rank test was used to compare the amount of transferred points in the trust and risk games for each group.

### Neuroimaging analyses

#### Functional analyses

In the video task, we modelled a parametric modulated regressor for each participant that included the 15 s that each financial transaction video lasted, weighted by the self-rated scores of safety (from 1 to 4) recorded by each participant. In accordance with our aims, we did not include the pleasant and unpleasant animals videos. We defined a positive contrast in which the higher activation represents a higher degree of security. This method allowed us to test whether the brain areas activated that are linked to the feeling of safety differ between groups while they watch any type of transaction.

In the trust and risk games, we modelled the brain activation during the decision phase, beginning when the message informed participants which game they were to play and up to the moment when they pressed the button indicating the amount they transferred. We defined two conditions (i.e., trust and risk) collapsing brain activation during trust or risk trials. Afterwards, we defined a trust > risk contrast of interest to explore the brain activation linked to participants’ trust. Task regressors were convolved with the SPM8 canonical hemodynamic function. To prevent motion artifacts, the six calculated parameters of movement, as well as the identities of the volumes labeled as outliers during preprocessing, were entered as regressors of no interest in all first-level analyses. Then, the individual first-level contrast images were used to conduct two one-sample ANOVA models to calculate patterns of activation within groups and between-group differences.

In order to restrict the analyses between groups to brain areas activated by the main effect of the task, the analyses between groups were masked by the sum of the maps of activation and deactivation derived from the corresponding one-sample analyses within groups. Using this method, we restrict the between-group results to brain areas linked to the feelings of safety or unsafety and trust for the videos and the trust and risk games, respectively.

A brain mask of 18,758 voxels resulting from the analyses within groups was used in the video task, whereas a voxels mask of 27,863 voxels that comprised the brain areas activated by the trust > risk contrast in both groups was used in the trust and risk games. Those masks mainly involved brain areas previously related to safety and trust processes, respectively (e.g., lateral prefrontal cortex, striatum, insula, and cingulum).

In order to ensure that the results are robust to multiple comparisons, we combined different thresholds of voxel intensity and cluster extent. We followed the SPM REST toolbox and used 1,000 Monte Carlo simulations to identify the spatial extent threshold. This was done using AlphaSim and following the SPM REST toolbox^42^. Regarding the inputs provided, taking into account the smoothness of data after model estimation, we included a mask corresponding to each task, an individual voxel threshold probability of 0.005, and a cluster connection radius of 5 mm. A minimum cluster-extent of 54 voxels and 59 voxels were estimated for the video task and the trust and risk games, respectively. Cohen’s d effect size was calculated for all regions showing significant differences between groups.

#### Structural analyses

For the T1 analyses, we explored differences in brain cortical and subcortical volumes between groups using one-way ANOVAs and two-sample group t-tests. Based on existing literature and our functional results, structural analyses were restricted to some regions of interest (i.e., frontal regions, striatum, insula, cingulum, amygdala, and hippocampus). Total intracranial volume was used as a confound variable in these analyses to control for the variability in participants’ head sizes^43^.

Regarding DTI analyses, we conducted one-way ANOVA’s, followed by between-group t-test comparisons in callosal fibers (i.e., forceps minor and major), limbic system fibers (i.e., cingulate gyrus and parahippocampal parts of the cingulum), association fibers (i.e., superior and inferior longitudinal fasciculus), and the corticospinal tract. ETA^2^ effect size was calculated for all regions showing significant differences between groups in all structural analyses.

#### Correlation analyses

Brain regions showing significant differences between groups in functional or structural analyses were correlated with behavioral scores from tests and fMRI tasks (i.e. security scores attached corresponding to traditional or new digital transactions represent the amount transferred during the risk and trust games). Pearson correlations were computed (using SPSS).

## Results

### Sample description

The groups did not differ significantly in terms of age, sex, employment, or monthly family income (See Table 1). Importantly, there were some consistencies across groups in terms of levels of financial knowledge and usage. First, most users had a bank account (90% in the NU group and 100% in the LFU and HFU groups). Second, the overwhelming majority of participants were aware of the online possibilities for their bank accounts (95% in the NU group and 100% in the other two groups). Third, the number of users of non-bank digital channels (e.g., Paypal) monotonically increased with the general frequency of use of bank digital channels (28% in the NU group, 43% in the LFU group, and 51% in the HFU group). The use of traditional payment methods was similar across groups (use of debit cards was 75% for the NU group, 86% for the LFU group, and 88% for the HFU group). Fourth, the variety in the use of digital financial transactions also increased monotonically with the frequency of use. In particular, none of the NU participants used more than three different digital services (i.e., check account balance, online bank transfer, online purchase, and bill payment), while 19% of the LFU group and 62% of the HFU group did. This suggests frequency of use and variety of use are highly correlated.

**Table 1:** Descriptive statistics of the study population grouped according to their frequency of use of digital financial services.

	Never (n = 41)	Low frequency (n = 40)	High frequency (n = 40)	*P* value	ETA^2^
Age	21.29 (2.90)	22.20 (3.15)	21.93 (2.53)	0.347	0.018
Sex	21 men	20 men	15 men	0.394	0.015
	20 women	20 women	25 women		
Workers	5 (12.20%)	12 (30%)	10 (25%)	0.139	0.033
**Family income**				0.250	0.014
< 600 €	3 (7.3%)	7 (17.5%)	1 (2.5%)		
600–1,000 €	7 (17.1%)	7 (17.5%)	6 (15.0%)		
1,000–1,500 €	12 (29.3%)	7 (17.5%)	9 (22.5%)		
1,500–2,000€	6 (14.6%)	6 (15.0%)	9 (22.5%)		
2,000–3,000€	10 (24.4%)	7 (17.5%)	10 (25.0%)		
3,000–5,000 €	3 (7.3%)	3 (7.5%)	5 (12.5%)		
> 5,000 €	0	3 (7.5%)	0		

Mean and (SD) values or n (%) are provided. *P* value represents the significance of the ANOVA analysis for this variable.

### Behavioral measures

#### Neuropsychological measurements

As shown in Table 2, we found no significant differences between groups in the ANOVA analyses of any of the tests (SPSRQ and UPPS-P).

**Table 2:** Results of the neuropsychological tests.

	All	Never (NU)	Low frequency (LFU)	High frequency (HFU)	*P* value	ETA^2^
**SPSRQ**						
Sensitivity to Reward	11.02 (4.09)	10.93 (3.76)	11.60 (4.53)	10.55 (3.97)	0.512	0.011
Sensitivity to Punishment	10.46 (5.46)	10.44 (5.66)	9.23 (4.89)	11.73 (5.62)	0.122	0.035
**UPPS-P**						
Negative Urgency	10.93 (2.94)	11.49 (3.12)	10.70 (2.76)	10.60 (2.90)	0.330	0.019
Positive Urgency	9.80 (2.08)	10.32 (2.52)	9.63 (2.00)	9.45 (1.89)	0.138	0.033
Lack of premeditation	7.81 (2.54)	7.46 (2.47)	8.15 (2.43)	7.82 (2.71)	0.479	0.012
Lack of perseverance	7.48 (2.64)	7.05 (2.66)	7.54 (2.48)	7.85 (2.76)	0.390	0.016
Sensation seeking	9.43 (2.41)	9.63 (2.29)	9.28 (2.55)	9.35 (2.43)	0.788	0.004

Mean and (SD) values are provided.

### fMRI behavioral measures

#### Video task

Security scores for each type of video are reported in Table 3. Between-group analyses did not show significant differences. Additionally, we computed the mean values for traditional financial transactions (i.e., cash, ATM, and card) and new digital transactions (i.e., online bank transfer, PayPal, phone, and watch) separately. No significant differences between groups were found for these variables (all |Z|< 1.6, *p* > 0.1 and ETA^2^ < 0.04).

**Table 3:** Security scores for all participants and by group.

	All	Never (NU)	Low frequency (LFU)	High frequency (HFU)
Cash	3.21 (0.68)	3.18 (0.60)	3.26 (0.70)	3.20 (0.74)
ATM	3.07 (0.70)	3.21 (0.51)	3.12 (0.78)	2.89 (0.77)
Card	2.82 (0.70)	2.92 (0.66)	2.77 (0.70)	2.76 (0.74)
Transfer	2.72 (0.79)	2.54 (0.80)	2.86 (0.81)	2.74 (0.75)
PayPal	2.64 (0.74)	2.58 (0.72)	2.67 (0.82)	2.67 (0.69)
Phone	2.24 (0.86)	2.16 (0.83)	2.40 (1.01)	2.15 (0.72)
Watch	2.03 (0.86)	1.99 (0.79)	2.14 (1.00)	1.97 (0.76)
Traditional	3.04 (0.43)	3.10 (0.39)	3.05 (0.41)	2.95 (0.47)
New digital	2.41 (0.61)	2.32 (0.60)	2.52 (0.67)	2.38 (0.56)

Mean and (SD) values are provided.

#### Trust and risk games

Table 4 shows the mean of transferred points by the three groups (NU, LFU, and HFU) in both the trust and risk games. In the trust game, a two-sample Mann–Whitney test showed that differences in transferred points between the groups were not statistically significant (Z = 0.718, *p* = 0.473, ETA^2^ = 0.007; Z = 0.381, *p* = 0.704, ETA^2^ = 0.002; and Z = − 0.482, *p* = 0.630, ETA^2^ = 0.003; for the comparisons between NU and LFU, NU and HFU, and LFU and HFU, respectively). For the risk game, a two-sample Mann–Whitney test showed that the NU group transferred less money than the LFU group (Z = − 1.991, *p* = 0.047, ETA^2^ = 0.050). Conversely, the differences between the LFU and the HFU groups and between the NU and HFU groups were not significant (Z = 0.121, *p* = 0.904, ETA^2^ = 0.0001 and Z = − 1.701, *p* = 0.089, ETA^2^ = 0.037, respectively).

**Table 4:** Mean of transferred points in the trust and risk trials.

	All	Never (NU)	Low frequency (LFU)	High frequency (HFU)
Trust	4.93 (2.37)	5.13 (2.53)	4.74 (2.50)	4.92 (2.10)
Risk	5.18 (1.95)	4.76 (1.76)	5.45 (2.04)	5.34 (2.01)
Betrayal aversion	0.25 (2.59)	− 0.37 (2.96)	0.71 (2.75)	0.42 (1.87)

Mean and (SD) values are provided.

Regarding betrayal aversion, a Wilcoxon signed-rank test showed no significant differences in the transferred points between games for the three groups (Z = 0.551, *p* = 0.581, ETA^2^ = 0.004; Z = − 1.540, *p* = 0.124, ETA^2^ = 0.030; and Z = − 1.406, *p* = 0.16, ETA^2^ = 0.025; for NU, LFU, and HFU, respectively).

### Neuroimaging results

#### Functional results

##### Video task

**Figure 1:**
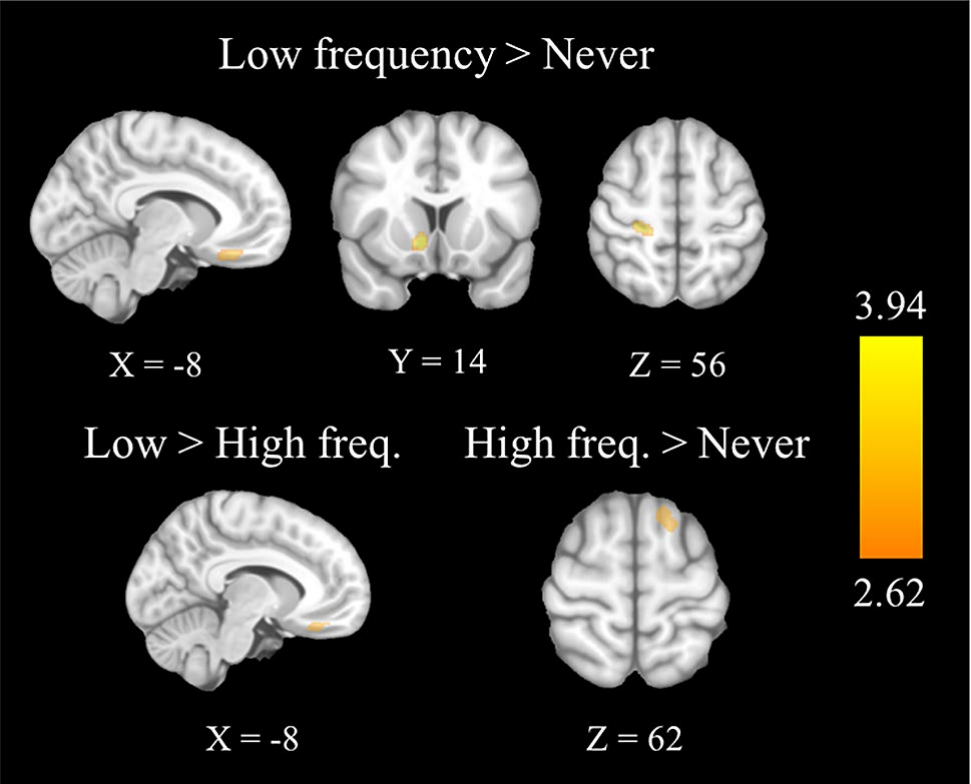
Brain regions showing differences between groups during the fMRI video task. Brain images display the differences between groups in brain activation between high frequency users, low frequency users, and those who never use online financial services while doing a fMRI video task. During this task, participants watched videos of traditional (e.g., paying with card) and new digital financial transactions (e.g., paying with a mobile phone) and reported the subjective feeling of safety they perceived. A parametric regressor model was used to estimate the brain activation linked to the feelings of security for each group. A one-way ANOVA model, followed by two-sample t-tests, was performed to compare groups two by two in all regions all regions on which the effect of the task was significant. All regions are significant at *p* < 0.005 with a cluster extent of 54 voxels. The right hemisphere is displayed on the left. The color bar indicates t-value. Image generated using SPM12 run under Matlab R2017 (https://www.fil.ion.ucl.ac.uk/spm/).

Comparison between groups revealed that the LFU group showed higher activation in the ventromedial prefrontal cortex compared to the other two groups. Additionally, the LFU group showed higher activation in the right precentral cortex and in the right nucleus accumbens in comparison with the NU group (see Table 5 and Fig. 1). Correlation analyses showed significant positive association between the brain activation of the nucleus accumbens and the mean scores of security of the new digital financial transaction videos in the LFU group (r = 0.489, *p* = 0.001), whereas this relationship was negative and insignificant in the HFU (r = − 0.182, *p* = 0.281) and NU groups (r = − 0.063, *p* = 0.701).

**Table 5:** Brain regions showing significant differences between groups during the video task.

Brain region	Side	MNI Coordinates			Cluster Size	t-value	z-value	Cohen’s d
		X	Y	Z				
**HFU > NU**								
Superior frontal Gyrus	Left	− 14	20	60	76	3.94	3.81	0.75
**LFU > NU**								
Ventromedial prefrontal Cortex	Left	− 10	28	− 22	79	3.76	3.64	0.71
Precentral Cortex	Right	24	− 28	56	63	3.41	3.32	0.64
Nucleus Accumbens	Right	10	10	− 8	54	3.34	3.26	0.63
**LFU > HFU**								
Ventromedial Prefrontal Cortex	Left	− 10	32	− 20	56	3.46	3.36	0.65

The HFU group showed higher activation in the superior frontal gyrus compared to the group that never used online financial services. Correlation analyses showed that brain activation in this area is negatively related to the security scores, so the higher the activation, the lower the feeling of security in all groups (r = − 0.267, *p* = 0.004) (see Table 5 and Fig. 1).

##### Trust and risk games

**Figure 2:**
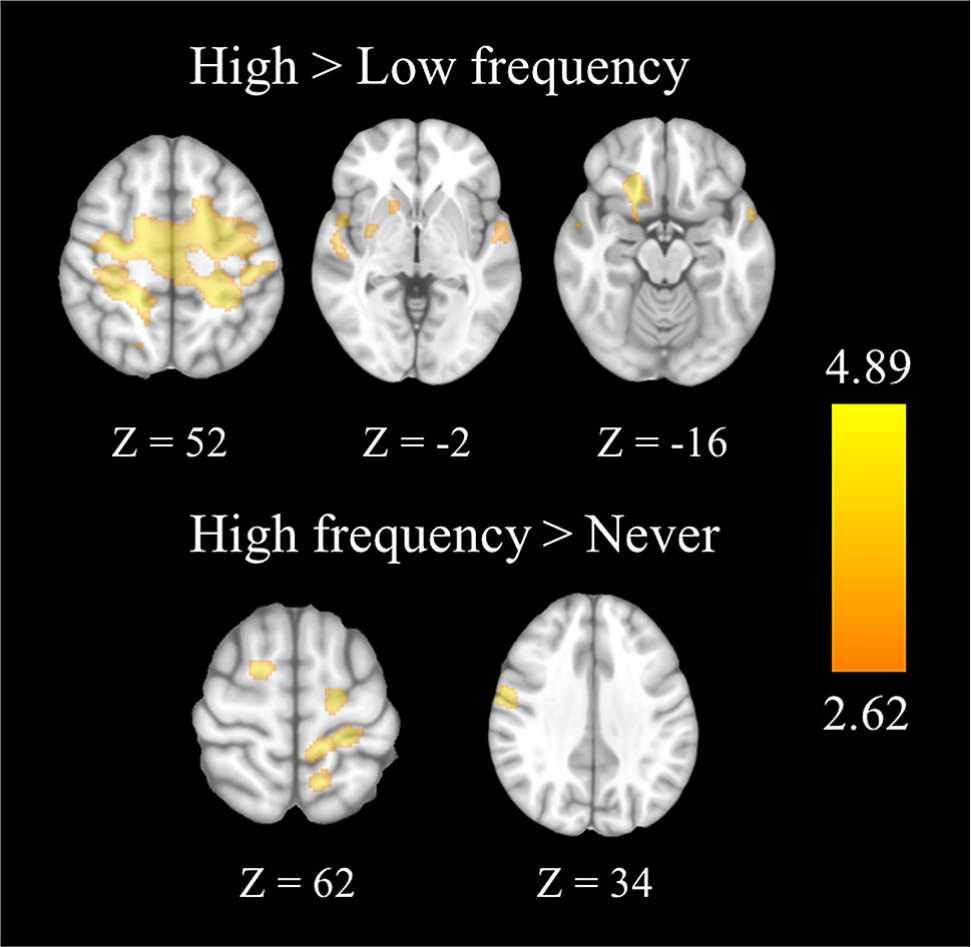
Brain regions showing differences between groups in the trust > risk contrast during the fMRI trust and risk tasks. Brain images display the differences between groups in brain activation between high frequency users, low frequency users, and those who never use online financial services while doing trust and risk games adapted to a fMRI environment. Participants performed several trials of both games, and brain activation was modelled during the decision phase of each one. Brain activation represents the differences in the defined trust > risk contrast. A one-way ANOVA model, followed by two-sample t-test, was performed to compare groups two by two in all regions on which the effect of the task was significant. All regions are significant at *p* < 0.005 with a cluster extent of 59 voxels. The right hemisphere is displayed on the left. The color bar indicates t-value. Image generated using SPM12 run under Matlab R2017 (https://www.fil.ion.ucl.ac.uk/spm/).

During the trust trials (Table 6 and Fig. 2)—in comparison with the risk trials—the HFU group showed higher activation of the precentral gyrus, postcentral gyrus, and supplementary motor area than the other two groups.

**Table 6:** Brain regions showing significant differences between groups during the trust and risk games.

Brain region	Side	MNI Coordinates			Cluster Size	t-value	z-value	Cohen’s d
		X	Y	Z				
**HFU > NU**								
Postcentral Gyrus	Left	− 22	− 36	54	334	3.82	3.70	0.72
Precentral Gyrus	Right	60	− 2	34	98	3.54	3.44	0.67
Pre-Supplementary Motor Area	Right	18	2	60	101	3.50	3.40	0.66
Precentral Gyrus	Left	− 36	− 12	50	117	3.48	3.39	0.66
Precentral Gyrus	Left	− 20	− 14	62	59	3.39	3.30	0.64
**HFU > LFU**								
Precentral Gyrus	Right	30	− 12	52	6,925*	4.89	4.65	0.92
Postcentral Gyrus	Left	− 24	− 38	52	6,925*	4.88	4.64	0.92
Supplementary Motor Area	R/L	8	− 22	58	6,925*	4.24	4.07	0.80
Putamen	Right	30	− 8	14	809*	4.71	4.49	0.89
Insula	Right	34	− 6	14	809*	4.59	4.38	0.86
Superior Temporal Gyrus	Right	56	2	− 12	809*	3.71	3.60	0.70
Orbitofrontal Cortex	Right	20	24	− 14	302*	4.38	4.20	0.82
Nucleus Accumbens	Right	18	10	− 4	302*	2.84	2.79	0.53
Superior Temporal Gyrus	Left	− 54	− 26	16	126	3.63	3.52	0.68
Insula	Left	− 30	− 8	14	117	3.62	3.51	0.68

*p < 0.005.

### Structural results

#### T1 results

Participants of the HFU group showed a higher volume of the left inferior frontal gyrus (orbital part) and the right transverse frontopolar cortex compared with those of the LFU (*p* = 0.018, ETA^2^ = 0.065, and *p* = 0.018, ETA^2^ = 0.064) and the NU groups (*p* = 0.024, ETA^2^ = 0.059, and *p* = 0.004, ETA^2^ = 0.112).

The LFU group showed lower volumes in the left paracentral cortex and the right precentral sulcus (inferior part) compared with the HFU (*p* = 0.043, ETA^2^ = 0.049, and *p* = 0.006, ETA^2^ = 0.087) and NU groups (*p* = 0.005, ETA^2^ = 0.101, and *p* = 0.091, ETA^2^ = 0.049).

**Figure 3:**
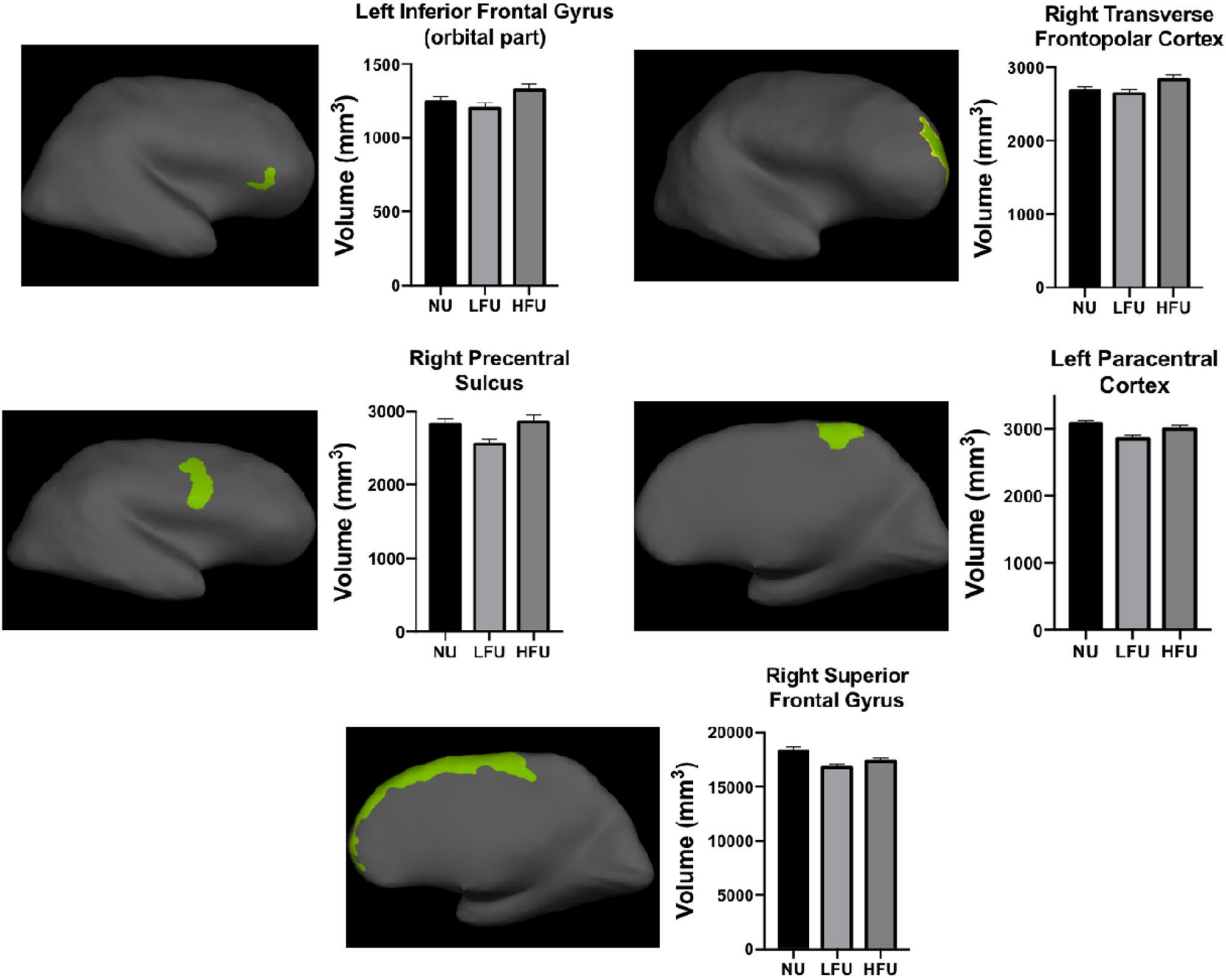
Brain regions showing differences in volume between groups in the structural analyses. A structural brain image of each participant was segmented in several regions, and the volume of each one was computed. Each image illustrates the location of the regions of the brain showing significant differences. Groups of participants that never use online financial services (NU), have a low frequency use (LFU), or have a high frequency use (HFU) are on the x-axis. The mean volumes of each brain region, measured in mm^3^, are on the y-axis. Image generated using Freesurfer (version 6.0) and GraphPad Prism (version 8.1.1).

Finally, the group of participants who never use online financial services showed higher volumes of the right superior frontal gyrus compared with LFU group (*p* = 0.008, ETA^2^ = 0.067), but not with the HFU group (*p* = 0.169, ETA^2^ = 0.026) (see Fig. 3).

#### DTI results

**Figure 4:**
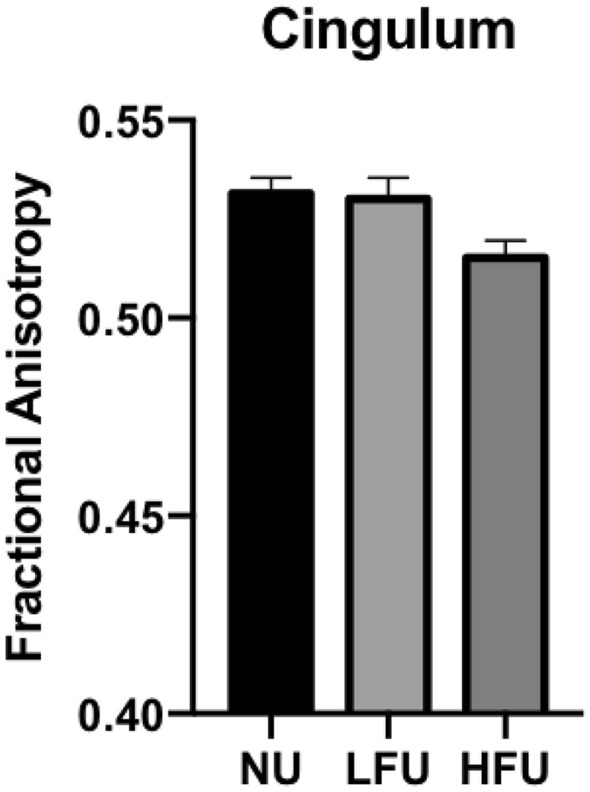
Fractional anisotropy values extracted from the cingulate gyrus part of the cingulum using diffusion tensor imaging (DTI). DTI images were preprocessed and probabilistic tractography were performed using AutoPtx for several tracts of interest. FA values were calculated for each of these tracts. Groups of participants that never use online financial services (NU), have a low frequency use (LFU), or have a high frequency use (HFU) are on the x-axis. Mean FA values of the cingulate gyrus part of the cingulum are on the y-axis. Image generated using Freesurfer (version 6.0) and GraphPad Prism (version 8.1.1).

The HFU group showed lower FA values in the cingulate gyrus part of the cingulum compared with the LFU group (*p* = 0.024, ETA^2^ = 0.056) and the NU group (*p* = 0.014, ETA^2^ = 0.088) (see Fig. 4).

No significant correlations were found between the brain volumes or the FA values and the behavioral variables.

## Discussion

The aim of this study is to explore the relationship between brain activity and trust and risk in digital financial behavior. The main finding is that brain activity reveals patterns around financial digitalization that have not been identified by any other economic or psychological tests. Impulsivity and sensitivity traits do not seem to explain differences in uses of financial digitalization, contrary to the first hypothesis, but there seems to be significant differences in the safety neural responses evoked during the video tasks, in line with the second hypothesis, and in brain volumes, in line with the third hypothesis.

In particular, we find that high frequency users of digital financial channels display enhanced motor, frontal, insular, and striatal activity linked to trust and increased regional volumes in frontal areas. They also show lower FA values in the cingulum. Conversely, low frequency users show higher prefrontal and striatal activity linked to security while watching financial transactions and lower volumes in motor brain regions. Regarding the video task, previous studies have linked the ventromedial prefrontal cortex activity to the processing of safety signals^44,45^. Furthermore, activation of the nucleus accumbens and also the vmPFC have been directly related to the processing of several rewarding stimuli^46^. Our results show higher activation of these areas in the case of low frequency users. Specifically, correlation analyses show a higher activation of the nucleus accumbens (related to higher safety feelings) while low frequency users are watching videos of financial transactions with new digital channels. Overall, these results indicate that, even in the absence of significant differences in safety scores after watching the videos, the LFU group shows a brain activation pattern of greater security and reward while watching the videos, specially while watching videos of new digital methods of payment. Regarding the other two groups, the HFU group shows a higher activation of the superior frontal gyrus in comparison with the NU group, but this activation is related to lower safety scores. Hence, the HFU group exhibits a brain pattern associated with lower safety during the video task. A possible interpretation of this result is that mobile phones, watches, or other new digital devices are frequently used to check balances or confirm that transfers and payments have been properly accounted for. The more transactions conducted, the higher the need for checking to avoid mistakes, overspending, or overdrafts. This potentially creates a spiral between the number of transactions and the need for confirmation and control of personal finances. This is connected to some extent with previous studies that suggest that trust and self-control have an impact on the use of electronic payment devices^47^. The findings are also connected to some extent with the results of shopping experiments that illustrate that there is more activation of brain regions connected to trust in less frequent (prudent) buyers than in more frequent and impulsive (hedonic) buyers^24^.

Interestingly, unlike the trust game—where we do not find significant differences in investment levels between the three groups—in the risk game the NU group shows a significantly lower willingness to invest when interacting with a random device in the role of trustee than the other two groups. Hence, contrary to the idea underlying the betrayal aversion concept, non-users seem to be less device-oriented than high and low frequency users^38,39^.

Moreover, during the trust and risk games, high frequency users show an enhanced somatomotor activation compared with the other two groups and specifically, enhanced insular, orbitofrontal, and striatal activity compared with low frequency users. Higher orbitofrontal and insular activity during the trust and risk games in the high frequency users is consistent with a previous study that shows higher activation in the OFC is related to fear and enhanced insular activity with distrust during financial transactions^14^. From this perspective, the HFU group exhibits an emotional decision-making pattern when they are faced with a trust-based decision-making. Moreover, the lower activation of reward and behavioral adaptation regions (e.g., dorsal striatum) in low frequency users is similar to results reported in a study in which participants received oxytocin during the trust game^10^. In that study, those who received oxytocin showed higher levels of trust and lower brain activation in similar areas than in our study. This is not surprising, because oxytocin is a neuropeptide that increases trust^36^, so the lower activity in the LFU group can be linked to higher levels of trust. Lastly, the higher activation of motor areas in the HFU group can be linked to previous results from another economic task, the ultimatum game. During this task, participants have to accept or reject fair and unfair monetary offers. Even when they lose something by rejecting, most people tend to reject unfair offers, showing an emotional processing of the situation. Higher activity in the brain motor areas has been consistently linked to rejecting unfair offers^48^. In our case, the similar pattern of higher activation in the motor areas in the HFU group may be associated with a similar emotional processing of the financial transactions.

No significant statistical relationships were found between differences in brain volume and the measured behavioral variables in our tests. Additionally, some significant results become insignificant when controlling for multiple comparisons. Future studies should further explore potential differences, perhaps using a region of interest approach, taking into account the preliminary results obtained in the present study. They should explore, for example, whether the higher inferior frontal gyrus volume in the HFU group is associated with higher response inhibition^49^, or if the lower volume in motor areas found in the LHU group could be associated with empathy and the mirror network^50^ or cognitive control^51^.

Additionally, fractional anisotropy is a measure of the microstructural integrity of the white matter, and lower values have been linked to axonal degeneration and demyelination. The cingulate gyrus is one of the main fiber bundles of the brain and connects many cortical and subcortical regions. Low FA values in the cingulum have been related to several pathologies and cognitive dysfunctions^52^, so the reduction in FA values in the HFU group seems to be related to less structural connectivity between distant brain areas. The lack of significant correlation between FA values and behavioral variables does not allow us to determinate how this alteration influences economic processing. Future studies should explore more in-depth whether the microstructure of this tract is in any way related to economic processing.

Finally, we do not find any behavioral difference between the groups in any of the impulsivity tests. General impulsivity traits do not explain the differences in the use of digital financial services, but according to our imaging results, feelings of security, or insecurity, and trust are better predictors of this different behavior.

In summary, lower safety neural responses evoked during the video task suggest that higher frequency of use of digital financial services could be linked to higher sensations of insecurity. Moreover, high frequency users show enhanced activation in brain areas linked to emotional processing during the trust game. These findings are relevant to decision-making for both public policy and private financial strategies. Many governments and multilateral institutions have assigned critical importance to exploring the patters of financial digitalization with the aim of promoting financial inclusion and reducing undesirable outcomes such as the shadow or informal economy. In the public arena, these attempts to promote financial inclusion through digitalization should consider these differences across individuals. In certain environments where financial inclusion can be promoted through digitalization (e.g., Africa and India), the relationship between trust and high-frequency use may have important consequences in terms of moving consumers from the informal to the formal economy. More generally, neural differences between high frequency and low frequency users may have implications for expenditure control and financial planning. On the private side, customer segmentation and relationship building by financial institutions should also consider these trust, security, and emotional patterns. Specifically, neuro indicators can be used for market segmentation, and the preferences of financial customers can be linked to behavioral outcomes such as evaluations of trustworthiness of digital channels.

This study has important strengths. To our knowledge, it is the first that explores neural substrates of online financial users and provides evidence of functional and structural brain differences relating to different patterns of use. Our findings of differences in brain structure and function are also supported by correlations between brain and behavioral outcomes. Furthermore, we obtained results using three different MRI techniques, including two fMRI tasks, using robust and well-established methodologies and analysis protocols. Finally, the groups were selected from a large database of potential participants and were well matched in sociodemographic characteristics. Among the avenues for further research, it would be interesting to split users up according to their use of online methods (e.g., bank, online payments, or PayPal) and how they use them (e.g., only to check their account or for other reasons). Future studies should take this into account and explore the neural basis of patterns of use of specific digital financial channels.

Nevertheless, these results should be understood in the context of some limitations. First, the absence of significant results in some of the analyses could be due a lack of statistical power. Future studies should use our results to calculate an adequate sample size. Second, we have focused our hypothesis on some psychological traits, but we have perhaps ignored other traits that would also explain financial behavior. That could explain, for example, the lack of correlation between our structural differences and psychological variables.
